# Two cases of catheters inserted from the femoral vein straying into the hepatic vein, possibly owing to a Eustachian valve: a case report

**DOI:** 10.1186/s40981-022-00544-1

**Published:** 2022-07-23

**Authors:** Yoshiaki Takise, Takuma Maeda, Hiroki Yonezawa, Kimito Minami, Masahiro Morinaga, Yoshihiko Ohnishi

**Affiliations:** Department of Anesthesiology, National Cerebral and Cardiovascular Center, 6-1 Kishibeshinmachi Suita, Osaka, 564-8565 Japan

**Keywords:** Eustachian valve (EV), Minimally invasive cardiac surgery (MICS), Extracorporeal membrane oxygenation (ECMO), Hepatic vein (HV)

## Abstract

**Background:**

In minimally invasive cardiac surgery (MICS) and extracorporeal membrane oxygenation (ECMO), a guidewire is inserted from the femoral vein (FV) into the right atrium. However, rarely, the guidewire or catheter strays into the hepatic vein (HV) because of the inferior vena cava (IVC)-HV angle. We report two cases in which a guidewire and venous cannula from the FV strayed into the HV, likely owing to a Eustachian valve.

**Case presentation:**

Both patients were women who underwent transesophageal echocardiography-guided FV cannulation. In case 1, a guidewire from the FV strayed into the HV owing to a Eustachian valve. In case 2, ECMO was established postoperatively. Transthoracic echocardiography confirmed the venous cannula had strayed into the HV. Computed tomography indicated IVC-HC angles of 129° (case 1) and 102° (case 2).

**Conclusion:**

A Eustachian valve can impede devices inserted from the FV and even allow them to stray into the HV.

**Supplementary Information:**

The online version contains supplementary material available at 10.1186/s40981-022-00544-1.

## Background

Venous catheters for extracorporeal circulation are often inserted from the femoral vein during minimally invasive cardiac surgery (MICS) and extracorporeal membrane oxygenation (ECMO). Although venous catheters may migrate into the hepatic vein with high frequency after surgical insertion from the right atrium [1, 2], they rarely stray into the hepatic vein (HV) because of the anatomical structure and blood flow. Misplacement of venous cannula into the HV may lead to complications such as insufficient venous drainage and vascular injury [1–3]. We describe two cases of a guidewire and a venous canula, respectively, straying into the HV from the FV, possibly because of a Eustachian valve which is a structure at the junction of the right atrium and IVC.

## Case presentation

### Case 1

**Fig. 1:**
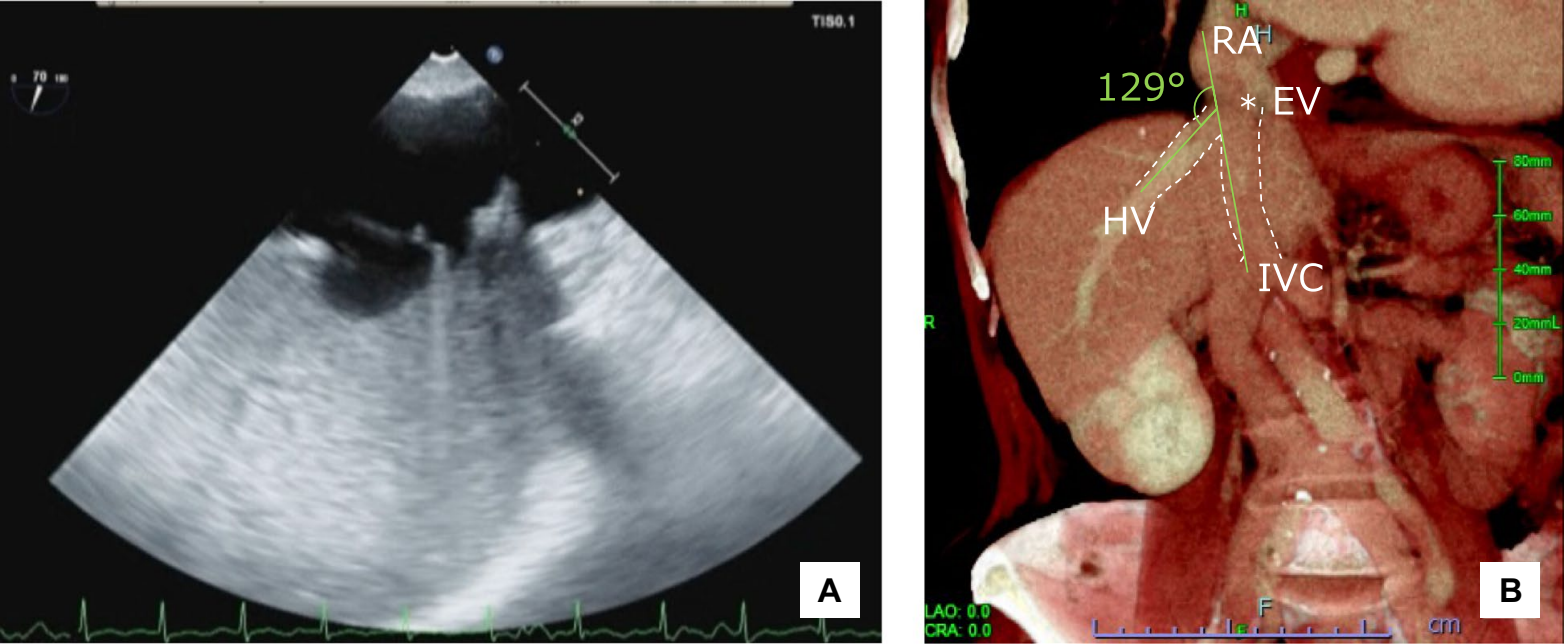
Echocardiographic and CT images in case 1 showing a strayed guidewire that was inserted from the FV to the HV. **A** TEE image of the IVC and HV junction. The GW was obstructed by the EV and strayed into the HV. **B** Coronal CT image of the junction of the HV and the IVC. The angle between the IVC and the HV is 129°, which is an obtuse angle. A low-absorption density indicating the Eustachian valve is seen on the right atrial side of the junction between the HV and the IVC. CT, computed tomography; FV, femoral vein; TEE, transesophageal echocardiography; ▼GW, guidewire; IVC, inferior vena cava; EV*, Eustachian valve; RA, right atrium; HV, hepatic vein

A 79-year-old woman (146 cm, 49.6 kg) was scheduled for minimally invasive mitral valve plasty for severe mitral regulation (MR). She had a history of hypertension, dyslipidemia, and paroxysmal atrial fibrillation. Preoperative transthoracic echocardiography (TTE) revealed severe MR due to prolapse of the posterior leaflet and moderate tricuspid regurgitation. No other structural abnormalities were observed. Preoperative computed tomography (CT) revealed no structural abnormalities, including skeletal abnormalities, such as scoliosis. Blood testing results were within normal limits. After induction of general anesthesia and tracheal intubation, a transesophageal echocardiography (TEE) probe was inserted. Extracorporeal circulation was scheduled to be established from the right femoral artery (FA) and right FV. Intraoperative TEE showed a hyperechoic membranous structure that appeared to be a Eustachian valve at the junction of the right atrium and IVC. To place the venous canula, a guidewire was inserted antegrade from the right FV into the right atrium; however, the Eustachian valve obstructed the guidewire, which strayed into the HV (Fig. 1A). The guidewire strayed into the HV several times, and it was very difficult to guide it into the right atrium. However, the direction of the wire was subsequently adjusted under TEE guidance, and the cannula was successfully inserted from the right FV (video). The postoperative reconstructed CT image confirmed the Eustachian valve and showed that the angle between the IVC and HV was 129° (Fig. 1B).

### Case 2

**Fig. 2:**
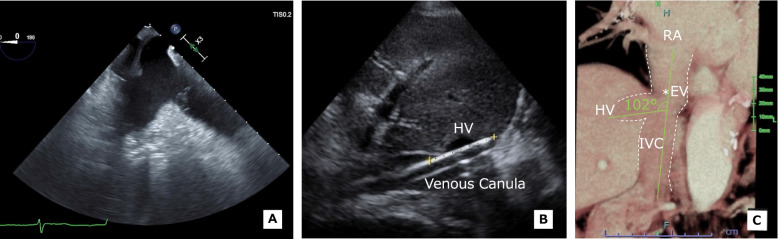
Echocardiographic and CT images in case 2 with a strayed venous canula that was inserted from the FV to the HV. **A** TEE image of the IVC and right atrial junction. A soft tissue density that is continuous with an obvious Eustachian valve is seen at the junction of the right atrium and IVC, near the coronary sinus. **B** TTE image showing that the venous canula strayed into the HV. **C** Coronal CT image of the junction of the HV and the IVC. The angle between the IVC and the HV is 102°, which is an obtuse angle. A low-absorption density indicating the Eustachian valve is seen on the right atrial side of the junction between the HV and the IVC. CT, computed tomography; FV, femoral vein; TEE, transesophageal echocardiography; IVC, inferior vena cava; EV*, Eustachian valve; RA, right atrium; HV, hepatic vein; RV, right ventricle; TV, tricuspid valve; TTE, transthoracic echocardiography

A 78-year-old woman (142 cm, 49.6 kg) was scheduled for aortic valve replacement (AVR) and total arch reconstruction (TAR) with an open stent graft for infective endocarditis and prosthetic valve endocarditis. She had a history of hypertension, previous AVR, and chronic kidney disease. Preoperative TTE revealed moderate MR owing to a severe coaptation defect, confirmed by abnormal echocardiography and which was a presumed abscess on the anterior mitral leaflet. Abnormal echocardiographic findings were also observed below the aortic valve as mobile structures attached to the subaortic curtain. No obvious anatomical abnormalities were observed in the right heart. Preoperative CT revealed left pleural effusion but no structural abnormalities. Blood testing results were within normal limits. After induction of general anesthesia in the operating room, TEE was performed. Extracorporeal circulation was scheduled to be established via the right subclavian artery and right FA with venous canulation of the right atrium from the right FV. The junction of the HV and IVC was difficult to observe, the tip of the venous cannula could not be seen, and a membrane-like structure connected to the Eustachian valve was observed at the junction of the right atrium and IVC (Fig. 2A). Extracorporeal circulation was uneventful, and AVR and TAR were completed as planned. However, bleeding was poorly controlled, and hemostatic manipulation was difficult. Therefore, the decision was made to establish ECMO and transfer the patient to the intensive care unit. TEE was performed after establishing ECMO, but the location of the tip of the venous catheter (Bio-Medicus NextGen Cannulae, 23 Fr) could not be confirmed, although the inside of the right atrium could be observed. Upon arrival of the patient in the intensive care unit, postoperative TTE confirmed that the venous cannula had strayed into the HV (Fig. 2B). The postoperative reconstructed CT image showed that the angle between the IVC and HV was 102° (Fig. 2C).

## Discussion

We experienced two patients in whom a guidewire and a venous canula, respectively, strayed into the HV when the devices were inserted through the FV, possibly caused by a Eustachian valve in each case.

The Eustachian valve is a strong membranous residual embryonic structure at the junction of the IVC and right atrium [4–6]. The average Eustachian valve coverage of the IVC orifice is approximately 23%, which usually does not interfere with catheter insertion, although there are reports of almost complete coverage in approximately 2% of cases [5]. This fetal structure can be an obstacle to catheter manipulation. Aung et al. [7] reported that the catheter was trapped by the Chiari network during pacemaker insertion in a heart failure patient, and Sarupria et al. [8] reported a case in which a venous cannula could not be inserted from the right atrium to the IVC owing to obstruction by a huge Eustachian valve. Ram et al. [9] also reported interference with the venous cannula in ECMO by the Chiari network and a Eustachian valve in the right atrium, resulting in a suck-down event. These findings were similar to our experience with the Eustachian valve obstructing the catheter.

Usually, a catheter is more likely to stray into the HV during insertion from the right atrium to the IVC because the angle between the IVC and the HV is acute in this direction. Therefore, it is assumed that catheter straying is less likely during insertion from the FV to the right atrium because the angle between the IVC and the HV is obtuse in this direction. In previous reports, guidewires inserted from the FV strayed into the ascending lumbar vein owing to the anatomical angle [10, 11]. This problem is thought to be related to the acute angle formed by the ascending lumbar vein and the iliac vein.

In case 1, the angle between the HV and IVC was 129°, which is an obtuse angle (Fig. 1B). However, a structure that appeared to be a Eustachian valve was observed on TEE (Fig. 1A) and CT (Fig. 1B) immediately adjacent to the junction of the IVC and HV. In case 2, the angle between the IVC and HV was also obtuse at 102° (Fig. 2B). As in case 1, a structure that appeared to be a Eustachian valve at the junction of the IVC and HV was observed on TEE (Fig. 2A) and CT (Fig. 2B). These observations indicate that a guidewire or venous catheter from the FV can stray into the HV even when the angle between the IVC and HV is obtuse.

Since this is a case report of two cases, it is unclear how obtuse the angle of guidewire straying is. However, if the angle is obtuse, it is considered anatomically difficult for a catheter to stray, unless there is a structure in the right atrium, such as a Eustachian valve. Our experience suggests that TEE guidance of guidewires and catheters into the right atrium and IVC is necessary, and that care must be taken to avoid complications from these devices straying into the HV when intra-atrial structures, such as the Eustachian valve, are present.

In conclusion, structures in the right atrium, such as the Eustachian valve, can obstruct the progress of a guidewire or venous canula from the FV and allow these devices to stray into the HV. Anesthesiologists must understand that in the presence of a right atrial structure, such as the Eustachian valve, a guidewire or venous canula may stray into the HV, even when the angle formed by the HV and IVC is obtuse.

## Supplementary Information

**Additional file 1.**

## Data Availability

The data in this case report are available from the corresponding author on reasonable request.

## Abbreviations

MICS: Minimally invasive cardiac surgery
ECMO: Extracorporeal membrane oxygenation
FA: Femoral artery
FV: Femoral vein
HV: Hepatic vein
IVC: Inferior vena cava
EV: Eustachian valve
TEE: Transesophageal echocardiography
TTE: Transthoracic echocardiography
CT: Computed tomography
AVR: Aortic valve replacement
TAR: Total arch reconstruction
